# The implications of subretinal fluid in pachychoroid neovasculopathy

**DOI:** 10.1038/s41598-021-83650-x

**Published:** 2021-02-18

**Authors:** Geun Woo Lee, Hyeon Cheol Roh, Se Woong Kang, A. Young Kim, Hoon Noh, Kyung Jun Choi

**Affiliations:** 1 Department of Ophthalmology, Samsung Medical Center, Sungkyunkwan University School of Medicine, Seoul, South Korea; 2 Department of Ophthalmology, Catholic University of Daegu School of Medicine, Daegu, South Korea

**Keywords:** Diseases, Eye diseases, Retinal diseases

## Abstract

This study aimed to identify the clinical characteristics and longitudinal changes in exudative pachychoroid neovasculopathy (PNV) and non-exudative PNV. This retrospective cohort study involved 81 eyes of PNV diagnosed by multimodal imaging including optical coherence tomography angiography. At baseline, they were divided into exudative PNV group and non-exudative PNV group depending on the presence of subretinal fluid. The clinical features of both groups and the longitudinal changes were investigated and compared. There were 55 eyes with non-exudative PNV and 26 eyes with exudative PNV. Individuals with non-exudative PNV were older, more frequently asymptomatic and had a higher prevalence of polypoidal choroidal vasculopathy in the opposite eye (all *P*’s < 0.05). Whereas individuals with exudative PNV showed thicker choroid and more frequent history of central serous chorioretinopathy (all *P*’s < 0.001). During about 12 months of longitudinal observation, the transformation into polypoidal choroidal vasculopathy was noted in 4 eyes of non-exudative PNV group, whereas in none of the exudative PNV group. Exudative PNV and non-exudative PNV seem to be separate entities with different epidemiological parameters. Non-exudative PNV, which is frequently found without symptoms at an older age, is suspected to be the significant precursor lesion of polypoidal choroidal vasculopathy. In contrast, exudative PNV may share the same pathophysiology as central serous chorioretinopathy.

## Introduction

Pachychoroid pigment epitheliopathy and pachychoroid neovasculopathy (PNV) are relatively recent diagnostic entities, and they share characteristics such as dilation of outer choroidal vessels and attenuation of the choriocapillaris (pachychoroid feature)^1–3^. Pachychoroid pigment epitheliopathy shows only abnormalities of retinal pigment epithelium (RPE) on top of the choroid with pachychoroid features, and PNV additionally presents type 1 (sub-RPE) choroidal neovascularization (CNV)^1,2^.

Since pachychoroid diseases may share the same mechanism, these can be linked with each other. Previous reports suggested that pachychoroid pigment epitheliopathy is likely to be a precursor of central serous chorioretiopathy (CSC), and long-standing CSC can induce type 1 CNV (i.e., PNV)^1–4^. And, silent pachychoroid pigment epitheliopathy may cause type1 CNV, thereby resulting in polypoid choroidal vasculopathy (PCV)^2,5^. There are, however, only a few reports on how pachychoroid diseases are linked to each other.

In this study, we divided PNV into exudative PNV and non-exudative PNV according to the presence of subretinal fluid at diagnosis. It was previously suspected that non-exudative and exudative PNV are part of the continuum of a single disease^3^. However, the presence of these two forms of PNV raises various questions. Do exudative PNVs come from non-exudative PNVs? Or are the non-exudative PNVs the end-stage form of exudative PNVs? Are they separate entities with different pathogenic implications and prognosis? They seem to be important from a clinical perspective as well as based on the pathomechanisms of pachychoroid spectrum disease. However, only a few reports have addressed these questions.

The purpose of this study was to identify and compare the clinical characteristics of non-exudative PNV and exudative PNV, and the longitudinal changes of the two entities.

## Methods

This retrospective cohort study was performed at a single center. This study approved by the Institutional Review Board of the Samsung Medical Center (IRB no. 2019-11-219), which waived the written informed consent because of the study’s retrospective design and was conducted in accordance with the tenets of the Declaration of Helsinki.

We retrospectively reviewed the medical records of patients with PNV. The diagnosis of PNV was confirmed when both pachychoroid features on optical coherence tomography (OCT) and type 1 CNV on optical coherence tomography angiography (OCTA) were observed without the phenotypes of age-related macular degeneration (AMD) such as soft drusen or related degenerative changes. The pachychoroid feature indicates the presence of pachyvessels with attenuation of the overlying sattler and choriocapillaris in the choroid. To sort out PCV, we excluded the cases with a suspected polyp on indocyanine green angiography or with sharp anterior protrusion of pigment epithelial detachment on OCT.

Among all patients diagnosed with PNV, we reviewed the information of the patients who were followed up so far. To be included in this study, all subjects were required to have undergone a comprehensive ophthalmologic examination, such as the measurements of best-corrected visual acuity (BCVA), manifest refraction, anterior segment examination using a slit lamp, dilated fundus examination, fundus photography, spectral-domain OCT (SPECTRALIS HRA-OCT; Heidelberg Engineering, Heidelberg, Germany), and swept-source OCTA (DRI OCT TRITON; Topcon Corporation, Tokyo, Japan). Visual acuity was measured using the Snellen chart. Fluorescein angiography and indocyanine green angiography (SPECTRALIS HRA-OCT; Heidelberg Engineering, Heidelberg, Germany) were used to distinguish PNV from PCV, typical AMD, and retinal angiomatous proliferation. Exclusion criteria comprised a spherical equivalent of more than − 6.0 diopters, a media opacity causing degradation in image quality and visual acuity, OCTA image quality index of less than 50, a history of ocular inflammation, a history of intraocular surgery other than cataract extraction, a history of ocular trauma or glaucoma, the presence of large pigment epithelial detachments (when a PCV is suspected).

The PNV patients were divided into the non-exudative PNV group and exudative PNV group according to the presence of subretinal fluid or other evidence of exudation at the time of PNV diagnosis (baseline). The chief complaints at baseline were categorized and compared between the two groups. The diagnoses in the fellow eyes were also compared.

In addition, longitudinal changes in each group were assessed. The eyes with non-exudative PNV which had manifested new exudation (exudative PNV or PCV) during the observation period were analyzed.

For the eyes with exudative PNV, the outcomes after treatment were analyzed. In the majority of exudative PNV, intravitreal anti-vascular endothelial growth factor (VEGF) treatment using a treat and extend regimen was the first-line treatment^6^. In the case of subfoveal fluid with a classic feature of CSC, but with the minimal extent of type 1 CNV, photodynamic therapy was attempted first. Half fluence photodynamic therapy was performed on the area of choroidal hyperpermeability that was identified by indocyanine green angiography^7^. In the case of only subtle exudation, the changes in visual acuity, symptoms, and exudation were closely monitored without treatment with a gradual increase in the follow-up interval.

**Figure 1:**
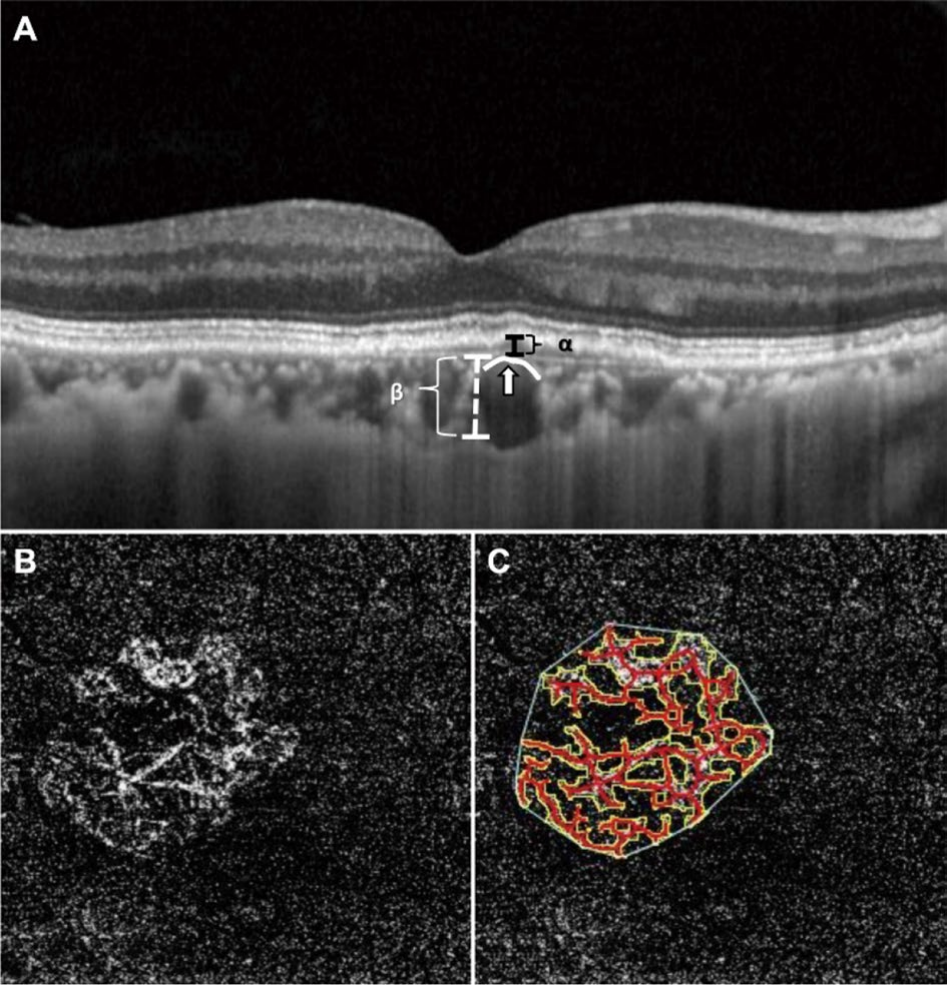
Measurements of choroidal thickness, DLS height, location of pachyvessel, CNV area, and CNV district. (**A**) DLS height (α) was defined as the highest among the raster scan of OCT (from Bruch membrane to outer border of the RPE layer). Subfoveal choroidal thickness (β) was the mean distance measured just below the fovea in the horizontal and vertical scans of OCT across the foveal center (from the Bruch membrane to sclerochoroidal junction). And, pachyvessel contour (white line) with attenuation of the choriocapillaris (white arrow) is seen below the DLS. (**B**) We obtained an en face image of OCTA that includes the CNV complex to the maximum extent possible. (**C**) ANGIOTOOL software was used to measure the extent of CNV district (the line surrounding the outer border of the entire CNV) and the area of CNV (the line surrounding the CNV). OCT, optical coherence tomography; OCTA, optical coherence tomography angiography; PNV, pachychoroid neovasculopathy; DLS, double-layer sign; CNV, choroidal neovascularization; RPE, retinal pigment epithelium.

The images of OCT and OCTA were acquired on every visit of all the PNV patients. OCT examination was performed in both the eyes using enhanced depth imaging mode. A 13-line horizontal raster scan was obtained in each eye covering the lesion at 30° × 10° with the horizontal and vertical scans passing through the center. The central retinal thickness was defined as the average thickness of the 1 mm circle of the foveal center. Subfoveal choroidal thickness was measured at the foveal center using the caliper function built into the OCT software. (Fig. 1A).

OCTA images that were automatically segmented in 3 × 3 mm^2^ macular regions were identified using viewing software. Two horizontal lines were adjusted to include the largest vessel complex in the en-face images of the 'outer retina' profile with CNV visible. The extent of CNV district and CNV area were measured using semi-automated and validated open-source software (ANGIOTOOL 0.6a, https://ccrod.cancer.gov/confluence/display/ROB2) (Fig. 1B, C).

Two examiners, who were blinded to all medical information, measured subfoveal choroidal thickness using OCT images (H.N., G.W.L.). And quantitive results of CNV using OCTA images were measured by two examiners blinded to all the medical information (A.Y.K., G.W.L.).

### Statistical analysis

BCVA was converted to the logMAR scale, and manifest refraction was converted to spherical equivalent for analyses. All continuous variables are reported as mean ± SD values. Shapiro–Wilk test was used for normality test. After that, the variables corresponding to the normal distribution were analyzed using the parametric method, and if the variables did not show a normal distribution, these were using the non-parametric method. Independent *t*-test and Mann–Whitney test were used to compare the continuous variables between the two groups. To compare the categorical variables between the two groups, the cross-tabulation analysis including the chi-square test and Fisher’s exact test was used. Longitudinal changes were compared with baseline and analyzed using the Wilcoxon signed-rank test. Statistical analyses were performed by SPSS version 25.0 for Windows (SPSS, Inc, Chicago, Illinois, USA) and R 3.6.1 (http://www.R-project.org, Vienna, Austria). All the reported *P* values less than 0.05 were considered significant.

## Results

A total of 81 eyes in 75 patients with PNV were enrolled in this study. At the time of diagnosis, 55 eyes belonged to the non-exudative PNV group and 26 eyes belonged to the exudative PNV group. The overall follow-up period for patients enrolled until October 2019 was an average of 12.2 ± 6.7 months. The mean follow-up period of exudative and non-exudative PNV was 12.7 ± 6.6 and 12.0 ± 6.8, respectively (*P* = 0.862).

Table 1 outlines the baseline demographic characteristics of PNV. Based on the gender, the proportion of males in total PNV was 71.6%, and there was no difference between the two groups. Non-exudative PNV had an older population than exudative PNV (62.7 ± 9.9 vs. 55.3 ± 15.5, *P* = 0.030). The mean logMAR BCVA of total PNV was 0.10 ± 0.17. Non-exudative PNV had better visual acuity than exudative PNV (0.03 ± 0.09 vs. 0.23 ± 0.21, *P* < 0.001). The patients with exudative PNV reported a more frequent history of CSC compared with the patients with non-exudative PNV (65.4% vs. 18.2%, *P* < 0.001).

**Table 1:** Baseline demographics.

Parameters	Total PNV (n = 81)	Exudative PNV (n = 26)	Non-exudative PNV (n = 55)	*P* value
Male: Female	58:23	17:9	41:14	0.393*
Age, years	60.4 ± 12.4	55.3 ± 15.5	62.7 ± 9.9	0.030^†^
BCVA, logMAR	0.10 ± 0.17	0.23 ± 0.21	0.03 ± 0.09	< 0.001*
IOP, mmHg	14.2 ± 2.7	0.14 ± 0.56	13.81 ± 0.35	0.064*
S.E, diopters	0.24 ± 1.34	0.07 ± 0.31	0.33 ± 0.17	0.860*
DM, n (%)	9 (11.1)	0 (0)	9 (16.4)	0.052^‡^
Hypertension, n (%)	25 (30.9)	7 (26.9)	18 (32.7)	0.620^§^
**Previous CSC episode**				
Number of eyes (%)	27 (33.3)	17 (65.4)	10 (18.2)	< 0.001^§^
The period from CSC episode to the diagnosis of PNV, months	22.8 ± 9.0	22.8 ± 9.6	23.0 ± 8.2	0.880*
Pseudophakia, n (%)	5 (6.2)	1 (3.8)	4 (7.3)	1.000^‡^

PNV, pachychoroid neovasculopathy; BCVA, best-corrected visual acuity; logMAR, logarithm of the minimum angle of resolution; S.E, Spherical equivalent; DM, Diabetes mellitus; CSC, Central serous chorioretinopathy.

Continuous variables are reported as mean value (± SD).

*Mann whiney test; ^†^Independent *t*-test; ^‡^Fisher's exact test; ^§^Chi-square test.

The subjects with exudative PNV visited the clinic with the complaint of visual disturbance such as blurred vision or metamorphopsia in 88.5% of cases. However, only 7.3% of cases with non-exudative PNV complained of visual disturbance. Most of the non-exudative PNVs were asymptomatic and were noted accidentally during routine eye examination as a part of a regular check-up or the disease in the fellow eye. The frequency of visual symptom was significantly different between the two groups (*P* < 0.001).

**Figure 2:**
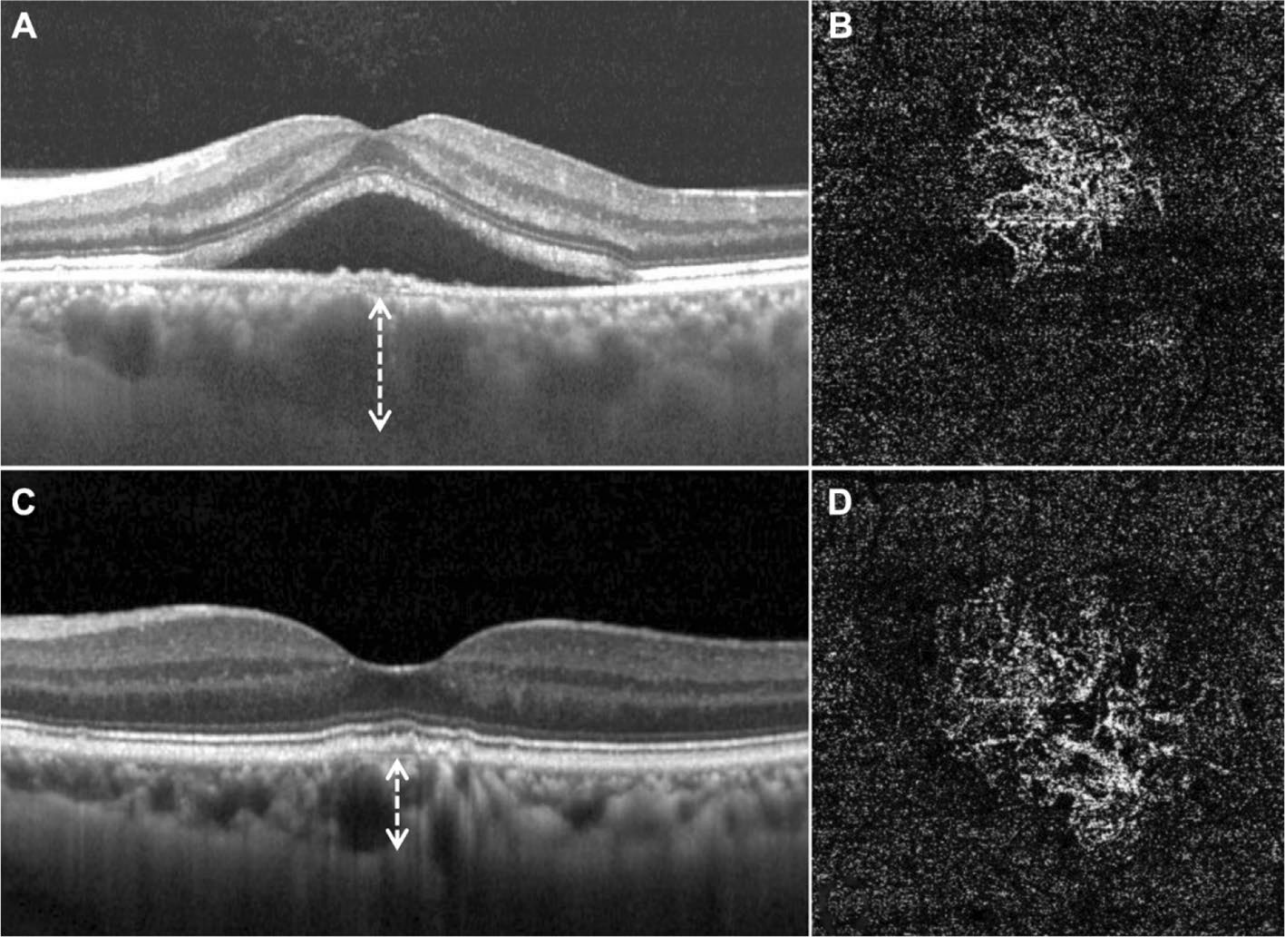
Representative OCT and OCTA images in the two groups. (**A**) and (**B**), Images of the left eye of a 58-year-old male with exudative PNV, who had a history of CSC. (**C**) and (**D**), Images of the left eye of a 66-year-old male with non-exudative PNV. (**A**) and (**C**), Exudative PNV has thicker choroid than non-exudative PNV (white dotted arrow, 422 μm, vs. 301 μm). (**B**) CNV image in DLS of exudative PNV. (**D**) CNV image in DLS of non-exudative PNV. Exudative PNV manifests higher vessel density. OCT, optical coherence tomography; OCTA, optical coherence tomography angiography; PNV, pachychoroid neovasculopathy; CNV, choroidal neovascularization; DLS, double layer sign.

Table 2 shows the comparisons of the baseline OCT and OCTA findings between the two groups. The average choroidal thickness in the total PNV group was 347.0 ± 45.1 μm, and the exudative PNV group had thicker choroid than the non-exudative PNV group (384.1 ± 38.3 vs. 329.5 ± 36.8, *P* < 0.001) (Fig. 2). Subfoveal choroidal thickness showed good agreement between the two examiners (mean intra-class correlation coefficient = 0.964 (95% confidence interval: 0.945–0.977)). The mean central retinal thickness of the total CNV was 291.4 ± 73.6 μm, which was significantly higher in the exudative PNV group than the non-exudative PNV group (332.4 ± 97.7 vs. 273.1 ± 48.8, *P* = 0.004). The mean extent of CNV district and area of CNV in total PNV cases were 0.91 ± 0.73 mm^2^ and 0.27 ± 0.19 mm^2^, respectively. There was no significant difference between the two groups. There was a good agreement between the two examiners in the extent of CNV district, and area of CNV (mean intra-class correlation coefficient = 0.995 (95% confidence interval: 0.992–0.996), 0.984 (0.976–0.990), respectively).

**Table 2:** Baseline features of OCT and OCTA.

Parameters	Total PNV (n = 81)	Exudative PNV (n = 26)	Non-exudative PNV (n = 55)	*P* value
Choroidal thickness, μm (Range)	347.0 ± 45.1 (235—476)	384.1 ± 38.3 (268—476)	329.5 ± 36.8 (235—412)	< 0.001*
**Diagnostic features**				
Pachychoroid only, n (%)	16 (22.2)	7 (26.9)	9 (16.4)	0.394^‡^
Pachyvessel only, n (%)	18 (19.8)	4 (15.4%)	14 (25.5)	
Both pachychoroid and pachyvessel, n (%)	47 (58.0)	15 (57.7)	32 (58.2)	
Central retinal thickness, μm	291.4 ± 73.6	332.4 ± 97.7	273.1 ± 48.8	0.004*
Extent of CNV district, mm^2^	0.78 ± 0.76	0.91 ± 0.73	0.75 ± 0.69	0.099*
Area of CNV, mm^2^	0.27 ± 0.19	0.25 ± 0.20	0.29 ± 0.19	0.199*

OCT, optical coherence tomography; OCTA, optical coherence tomography angiography; PNV, pachychoroid neovasculopathy; CNV, choroidal neovascularization.

Continuous variables are reported as mean value (± SD).

*Mann whiney test; ^†^Independent *t*-test; ^‡^chi-square test.

**Figure 3:**
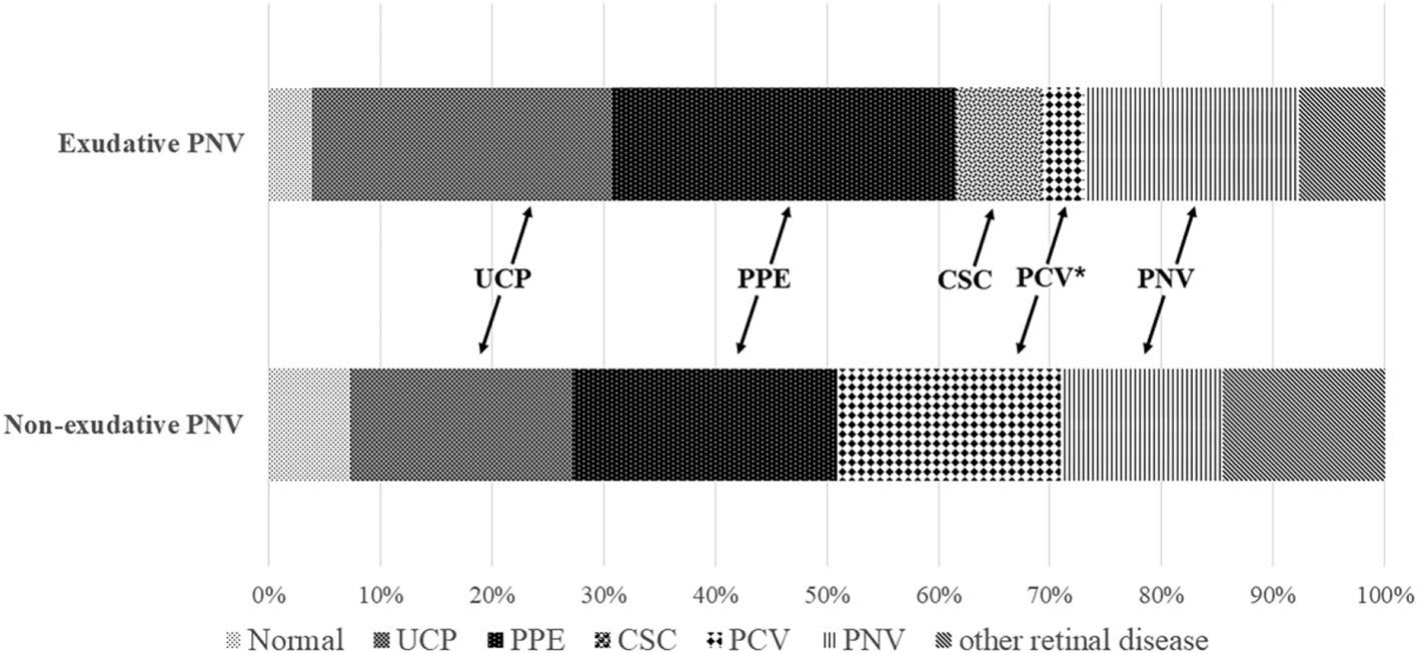
Distribution of the diagnosis in the fellow eyes. The only diagnosis with significant differences between the two groups was PCV (asterisk (*), 3.8% vs. 20.0%, P < 0.027, Fisher's exact test). UCP, uncomplicated pachychoroid; PPE, pachychoroid pigment epitheliopathy; CSC, central serous chorioretinopathy; PCV, polypoidal choroidal vasculopathy; PNV, pachychoroid neovasculopathy.

Figure 3 shows the distribution of diagnosis in the fellow eyes at baseline. PCV was more frequently noted in the fellow eyes of the non-exudative PNV group than those of the exudative PNV group (20.0% vs. 3.8%, *P* = 0.034).

**Figure 4:**
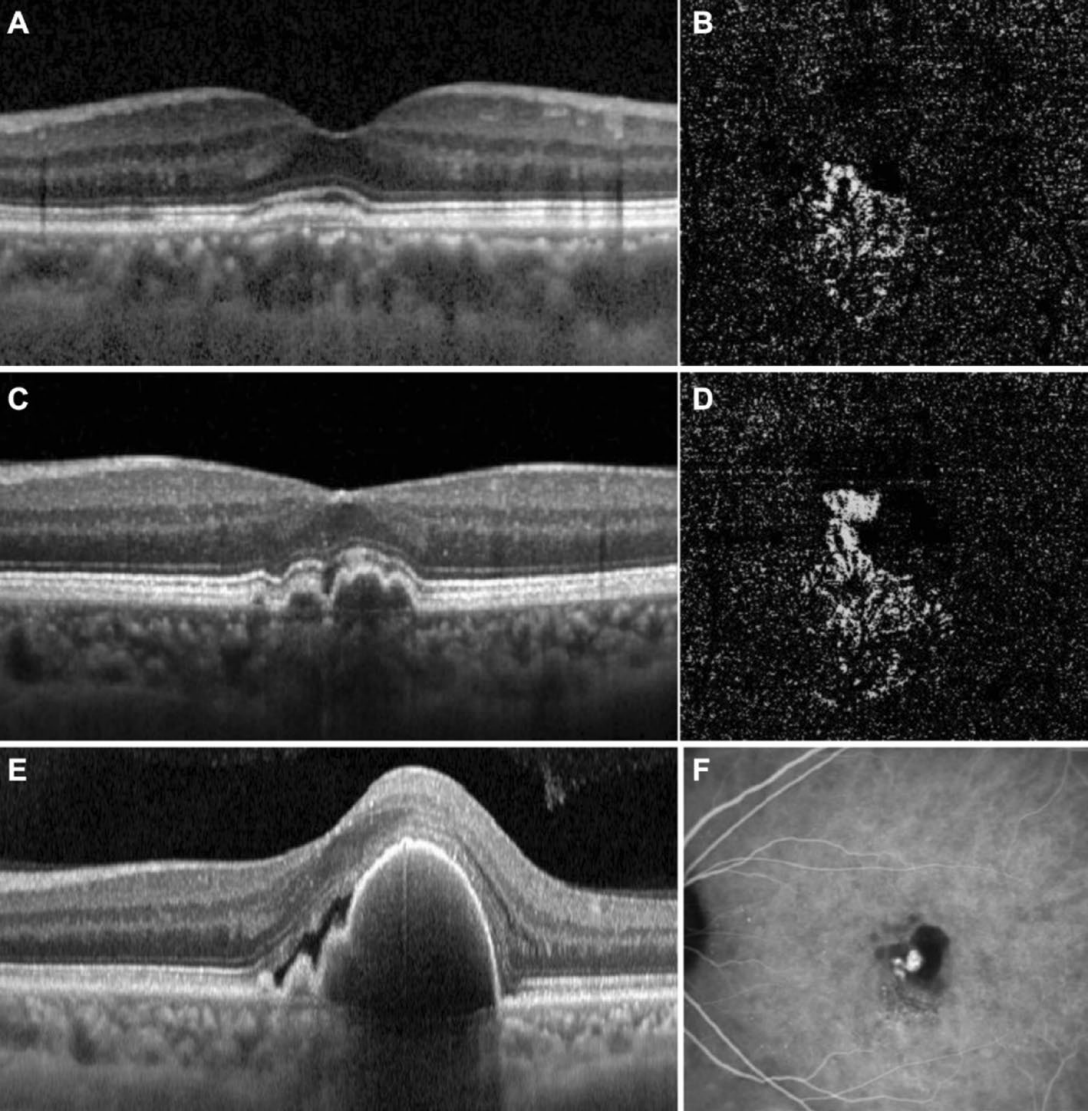
Images showing a temporal change in the images of the left eye of a 69-year-old male, in which non-exudative PNV transformed to PCV. (**A** and **B**), OCT and OCTA images of non-exudative PNV at baseline. There is no protruding RPED corresponding to a polyp, and there is no exudative change, such as subretinal fluid or intraretinal cyst formation, other than DLS. (**C** and **D**), Images taken 8 months after baseline. Note the increased height of the RPED on structural OCT and the enlargement of the extent of MNV on OCTA. (**E** and **F**), OCT and ICGA images after transformation to PCV 14 months after baseline. ICGA image demonstrates polyps and branching vascular networks. PNV, pachychoroid neovasculopathy; PCV, polypoidal choroidal vasculopathy; OCT, optical coherence tomography; OCTA, optical coherence tomography angiography; DLS, double layer sign; RPED, retinal pigment epithelial detachment ; MNV, macular neovascularization ;ICGA, indocyanine green angiography.

The longitudinal changes in each case were assessed. During the follow-up period, transformation to PCV was not found in exudative PNVs. On the contrary, five eyes among the non-exudative PNV group at the baseline had newly manifested exudation. One of them transformed to exudative PNV and the other four developed PCV (Fig. 4).

Table 3 shows changes in visual acuity, and the status of subretinal fluid according to the treatment patterns in exudative PNV group. In the anti-VEGF only group and the photodynamic therapy following anti-VEGF group, the average number of injection treatments was 5.6 ± 2.6 times and 6.0 ± 4.6, respectively. The degree of subretinal fluid compared with the baseline in the latest follow-up was divided into four categories; complete absorption, decreasing, stationary or increasing, and wax and wane. Overall, 13 eyes (50.0%) showed ‘complete absorption’ of fluid with and without treatment, and 9 eyes (34.6%) and 4 eyes (15.4%) showed ‘decreasing’ and ‘wax and wane’ pattern of subretinal fluid. Among respective treatment patterns, 7 eyes (41.2%) in the anti-VEGF only group, 4 eyes (100%) in the photodynamic therapy following anti-VEGF group, and 2 eyes (66.7%) in the observation group showed complete absorption of subretinal fluid. The BCVA after complete absorption of exudation in the 13 eyes was still significantly poor, compared with that of 55 eyes in the non-exudative PNV group (0.16 ± 0.20 vs. 0.03 ± 0.09, *P* = 0.006).

**Table 3:** Longitudinal changes in the exudative PNV group according to the treatment patterns.

Parameters	Total exudative PNV (n = 26)	Anti-VEGF (n = 17)	Anti-VEGF + PDT (n = 4)	PDT (n = 2)	Observation (n = 3)
**BCVA, logMAR**					
Baseline	0.23 ± 0.21	0.22 ± 0.23	0.29 ± 0.18	0.02 ± 0.03	0.30 ± 0.35
Latest follow up	0.15 ± 0.20	0.15 ± 0.21	0.14 ± 0.11	0.03 ± 0.04	0.32 ± 0.33
*P* value*	0.043	0.105	0.068	0.317	0.109
**SRF status compared with baseline, n (%)**					
Complete absorption	13 (50.0)	7 (41.2)	4 (100)	0 (0)	2 (66.7)
Decreasing	9 (34.6)	9 (52.9)	0 (0)	0 (0)	0 (0)
Stationary or increasing	0 (0)	0 (0)	0 (0)	0 (0)	0 (0)
Wax and wane	4 (15.4)	1 (5.9)	0 (0)	2 (100)	1 (33.3)
*P* value^†^	0.006				

PNV, pachychoroid neovasculopathy; VEGF, vascular endothelial growth factor; PDT = photodynamic therapy; BCVA = best-corrected visual acuity; logMAR = logarithm of the minimum angle of resolution; SRF = subretinal fluid.

Continuous variables are reported as mean value (± SD).

*Wilcoxon signed-rank test; ^†^Fisher's exact test.

## Discussion

OCTA demonstrated greater sensitivity in detecting type 1 CNV than conventional dye angiography in cases with pachychoroid spectrum disease^8^. Therefore, it is hypothesized that before the introduction of OCTA, most cases of exudative PNV might have been diagnosed with CSC in the past^8,9^. Until now, most of the reports regarding PNV have dealt with the eyes accompanying subretinal fluid^2,10–12^. The earlier reports regarding the pachychoroid spectrum suggested that the CSC may develop into exudative PNV, and PNV has been postulated as a possible precursor of PCV^10,13^. Also, recently, Matsumoto et al. reported that there is a clear anteroposterior relationship between age and central choroidal thickness among CSC, PNV, and PCV patients^14^. Only a few reports revealed that type 1 neovascularization might also occur in a more benign or ‘‘quiescent’’ form^4,15,16^.

As the main result of this study, first, the patients with non-exudative PNV were older than patients with exudative PNV. Most of the patients were found to be asymptomatic and had better visual acuity. They had a higher prevalence of PCV in the opposite eye, although this finding may be caused by the difference in mean age between the two groups. On the contrary, exudative PNV occurred in younger people with thicker choroid and more frequently had a history of CSC. Apart from the worse visual acuity at diagnosis in exudative PNV due to exudation, the significantly worse visual acuity was observed even after absorption of exudation compared with non-exudative PNV.

Second, this study addressed the approximate distribution of non-exudative PNV and exudative PNV among all observed cases of PNV. Although the proportion of non-exudative PNV seems to be no less than that of exudative PNV, this issue has not been addressed in previous literature. Non-exudative PNV is mostly asymptomatic and patients retain good visual acuity. Thus, it is discovered only rarely in the clinic and its prevalence seems to be underestimated. In contrast, it is possible that the frequency of non-exudative PNV, which is frequently seen in the opposite eye of PCVs, has been overestimated because of selection bias in the tertiary hospital patient population. Because of this inherent limitation of hospital-based studies, the relative frequency of the two conditions will not be accurate. A population-based study would provide the correct frequencies of the two conditions.

Third, this study also addressed the outcome of follow-up in non-exudative PNV cases and the prognosis of treatment in exudative PNV cases. Among the 55 eyes of the non-exudative PNV group, 5 eyes transformed into PCV (4 eyes) and exudative PNV (1 eye) during an average follow-up period of 12.0 ± 6.8 months. On the contrary, we were not able to detect PCV during follow-up of eyes with exudative PNV. This finding, along with the observation that PCV was noted more frequently in the fellow eye of the non-exudative PNV group, probably highlights the close linkage between non-exudative PNV and the development of PCV. In support of the results of the current study, Yanagi et al. reported the presence of a non-exudative type of PNV in the fellow eyes of patients with PCV^17^. The presence of pachychoroid pigment epitheliopathy seemed like a risk factor for such “silent” lesions.

We believe that the pathophysiologic component of CSC may, at least partly, contribute to the development of serous subretinal fluid in exudative PNV. If this is the case, the response to anti-VEGF treatment would be poor^18^. Indeed, of the 21 eyes treated with anti-VEGF as first-line treatment, only 7 eyes (33.3%) showed complete absorption of exudation. On the contrary, all 4 patients with poor response to anti-VEGF showed complete absorption of exudation after PDT, suggesting that this treatment response is similar to the response after CSC treatment^18^. One study revealed that the aqueous humor concentration of VEGF in PNV was lower than that in neovascular AMD^19^. These points also explain the poor treatment response to anti-VEGF and propose that exudative PNV may share the pathophysiology of CSC. Recently, Schworm et al. reported that neovascular CSCs may require more injection treatment than neovascular AMD because of the lower therapeutic response to anti-VEGF treatment, which is consistent with our results^20^.

Our results may defy the hypothesis that one type of PNV is the chronic form of the other type of PNV. Most non-exudative PNV is considered not to be transformed into exudative PNV. The primary basis for this assumption is the age of both groups. The progression from non-exudative PNV characterized by old age to exudative PNV characterized by a relatively younger age signifies a situation aging backward. Second, the results of longitudinal observation also support the assumption. Among non-exudative PNV in 55 eyes, only 1 eye transformed to exudative PNV during the follow-up period. Thus, although non-exudative PNV may rarely transform into exudative PNV, it will mainly remain silent or cause PCV.

It is also estimated that the majority of non-exudative PNV is not derived from exudative PNV. It is because the visual acuity after complete resolution of exudation by treatment in exudative PNV was still significantly worse compared with non-exudative PNV. Explicitly, it is difficult to expect that visual acuity in the eyes with permanent damage to neural tissue could improve significantly after several years. It is highly probable that a majority of non-exudative PNV would have occurred de novo without exudative episodes in the past.

In exudative PNV, cases of conversion to PCV after treatment are not infrequent. However, in most PCV patients in the real world, the history of PNV w/wo exudation is unknown. In other words, the cases of PNV with exudation that transitioned to PCV after treatment are estimated to represent the minority of all cases of PCV. One of the important implications of this study is that there are a substantial number of cases of asymptomatic non-exudative PNV. In addition, this study suggests that, despite the short follow-up period, PCV will occur later in a significant proportion of cases of non-exudative PNV.

The results of the current study suggest that non-exudative PNV, which is more intimately related to PCV than exudative PNV, might be a representative precursor lesion of PCV. Previous studies have also suggested the possibilities that “double-layer sign”, “flat irregular PED”, “RPE undulation”, and “late geographic hyperfluorescence” would be the precursor lesion of PCV^2,15,21–24^. They are the terminologies that describe the angiographic or tomographic manifestations of non-exudative PNV.

There are several limitations to this study. It was designed retrospectively and the follow-up period was relatively short. If we were able to observe a larger number of subjects over a longer period of time, we expect that we would gather evidence that would further support the results of this study. Also, it is unclear whether conversion to PCV is less due to the therapeutic effect of exudative PNV, and further research into this question is required. In addition, because non-exudative PNV was asymptomatic and often identified by chance, it is hard to capture the real prevalence and hazard of progression of the entity in this hospital base study. However, our study may provide new insights regarding the classification of NV and its pathogenic and prognostic significance.

To summarize, the patients with exudative PNV were relatively young, had a thick choroid, and visual symptoms and CSC history were more frequent (up to 66%). In contrast, the patients with non-exudative PNV were older but had better visual acuity and were asymptomatic without a history of exudation such as CSC. The frequent encounters with PCV in the fellow eyes or during the follow-up of non-exudative PNV suggest that this silent form of PNV could be the precursor lesion or the forme fruste of PCV.

In conclusion, PNV can be classified into two distinct types depending on the presence or absence of exudation at the time of diagnosis. Exudative PNV and non-exudative PNV seem to be separate entities with different epidemiological parameters. Exudative PNV may share the same pathophysiology as CSC. In contrast, non-exudative PNV, which is found without any symptoms at an older age, is likely to be the major precursor lesion of PCV.

## Data Availability

The data used to support the findings of this study are available from the corresponding author upon request.
